# A systematic review and meta-analysis on *Staphylococcus aureus* carriage in psoriasis, acne and rosacea

**DOI:** 10.1007/s10096-016-2647-3

**Published:** 2016-05-05

**Authors:** J. E. E. Totté, W. T. van der Feltz, L. G. M. Bode, A. van Belkum, E. J. van Zuuren, S. G. M. A. Pasmans

**Affiliations:** Department of Dermatology, Erasmus MC University Medical Center, Rotterdam, The Netherlands; Molecular and Cellular Life Sciences, Utrecht University, Utrecht, The Netherlands; Department of Medical Microbiology, University Medical Center Utrecht, Utrecht, The Netherlands; bioMérieux, Scientific Office, La Balme Les Grottes, France; Department of Medical Microbiology and Infectious Diseases, Erasmus MC University Medical Center, Rotterdam, The Netherlands; Department of Dermatology, Leiden University Medical Center, Leiden, The Netherlands

## Abstract

*Staphylococcus aureus* might amplify symptoms in chronic inflammatory skin diseases. This study evaluates skin and mucosal colonization with *S. aureus* in patients with psoriasis, acne and rosacea. A systematic literature search was conducted. Both odds ratios (OR) for colonization in patients versus controls and the prevalence of colonization in patients are reported. Fifteen articles about psoriasis and 13 about acne (12 having a control group) were included. No study in rosacea met our inclusion criteria. For psoriasis, one study out of three controlled studies showed increased skin colonization (OR 18.86; 95 % confidence interval [CI] 2.20–161.99). Three out of the five studies that reported on nasal colonization showed significant ORs varying from 1.73 (95 % CI 1.16–2.58) to 14.64 (95 % CI 2.82–75.95). For acne one of the three studies that evaluated skin colonization reported a significant OR of 4.16 (95 % CI 1.74–9.94). A relation between nasal colonization and acne was not found. Limitations in study design and low sample sizes should be taken into consideration when interpreting the results. Colonisation with *S. aureus* seems to be increased in patients with psoriasis. This bacterial species, known for its potential to induce long-lasting inflammation, might be involved in psoriasis pathogenesis. Information on acne is limited. Prospective controlled studies should further investigate the role of *S. aureus* in chronic inflammatory skin diseases.

**Electronic supplementary material:**

The online version of this article (doi:10.1007/s10096-016-2647-3) contains supplementary material, which is available to authorized users.

## Introduction

The inflammatory skin disorders atopic dermatitis, psoriasis, acne and rosacea have been associated with imbalances of the skin microbiome [1–4]. Local expansion of microbes with enhanced inflammatory potential, such as *Staphylococcus aureus*, was described as a potential mechanism for (secondary) inflammation in skin diseases [1]. In atopic dermatitis, patients are more likely to be colonized with *S. aureus* than healthy controls and colonization increases with AD severity [5]. Recent studies suggest that *S. aureus* contributes to long-lasting cutaneous inflammation in AD via binding to Toll-like receptor (TLR) 2 and local immunosuppression favoring prolonged colonization. This raises the question whether *S. aureus* might also play a role in other chronic inflammatory skin conditions, such as psoriasis, acne and rosacea [6].

Current research into the microbial pathogenesis of psoriasis focusses mainly on *Streptococci. Propionibacterium acnes* is most described in acne and *Demodex* mites are linked to rosacea [7–10]. However, the exact role of these microbes in the diseases is debated. In psoriasis, lesional skin is enriched in *Streptococcus* spp. [11]. However, evidence for the induction of the disease by a preceding streptococcal infection exist only for the subtype of guttate psoriasis [12]. *S. aureus* has been linked to exacerbations in psoriasis [7, 13]. It can activate Th1 and Th17 cells, promoting the production of interleukins TNF-α and IFN-γ which perpetuates keratinocyte damage [14–18]. Furthermore, staphylococcal enterotoxins can activate T cells inducing a more systemic immunological response, and staphylococcal peptidoglycan can favour proliferation of keratinocytes [19–22].

In acne, *Propionibacterium acnes* probably contributes to inflammation via stimulation of TLR2 [23–26]. However, certain strains rather than the entire species seem to be involved and the association between colonization and acne symptoms has been contested [27–29]. Chitin released from *Demodex* mites is thought to stimulate TLR2 receptors in rosacea [8, 9, 30, 31]. A role for *S. aureus* in acne and rosacea can be hypothesized based on *S. aureus*’ ability to stimulate TLR2, for example via alpha toxin [32–34].

Currently, antibiotics are part of standard therapy against acne and rosacea. Antibiotics often display broad spectrum activity which could damage the skin [35–37]. Furthermore, resistance against antibiotics is increasing, putting pressure on the (maintenance) treatment of inflammatory skin diseases [38–41]. In the development of new antimicrobial therapies, targeted treatment directed against a single bacterial species is gaining in importance [42]. In order to determine the scope of these new therapeutics we have to understand which specific microbes play a role in diseases. Investigating the abundance of *S. aureus* in a disease-specific ecosystem might give more insight in its possible role in pathogenesis. This systematic review and meta-analysis evaluates colonization of the skin and mucosa with *S. aureus* in patients with psoriasis, acne and rosacea compared with healthy controls.

## Materials and methods

### Type of study

Original experimental and observational (human) studies were included. Case reports were excluded. No restrictions were made as to publication date and language.

### Type of participants

Studies conducted in patients with psoriasis, acne or rosacea as diagnosed by a physician, were included. No restriction was made as to age of the patients.

### Type of outcome measures

The primary outcomes were proportion of patients with presence of *S. aureus* on the skin, nares or pharynx and the odds for colonization compared with healthy controls. The secondary outcome was presence of *S. aureus* virulence factors on the skin or encoded in the bacterial genome. In case of intervention studies, both pre- and post treatment measurements were included in this review, but only the baseline measurement was included in the meta-analysis. When studies reported multiple measurements over time taken from the same skin site (without treatment regimen) or when multiple locations were sampled at the same time point, the mean was included in the meta-analysis. Articles that reported combined results of different sample locations were excluded.

### Search strategy

The search was conducted in Embase, Medline, Ovid-SP, Pubmed, Web of Science and the Cochrane Central Database from inception to September 2014 (Table S1). A cross reference check was performed to identify further relevant studies.

### Study selection and data extraction

The titles and abstracts were screened for relevance and selected on the basis of the in- and exclusion criteria. The quality of the articles was rated using an extended version of the Newcastle Ottawa Scale (NOS) (Supplementary material) [43, 44]. Uncontrolled studies could reach a maximum score of 7 points for study quality on the NOS. Studies including a control group could reach a maximum score of 8. Using a scoring algorithm (Supplementary material), the controlled articles were classified as being of poor, fair or good quality [45]. Study selection and quality assessment were performed independently by two researchers (JT and WF). Disagreements were discussed and resolved. If identical populations were described in different publications (co-publication of same study), the study providing the most data was included.

### Statistical analysis

A weighted prevalence of colonization with *S. aureus* in the nose and/or skin was calculated. In controlled studies, the prevalence of colonization was compared between patients and controls and expressed as an odds ratio with a 95 % confidence interval. If possible, a meta-analysis was carried out using a random-effects model. Only studies in which patients were not receiving treatment were included for meta-analysis. Heterogeneity was assessed using the I^2^ statistic. If heterogeneity was < 80 %, data were pooled. The low number of studies did not permit an assessment of publication bias using funnel plots and Egger’s regression [46]. All statistical analyses were performed using Comprehensive Meta-Analysis Version 2.2 (Biostat, Englewood, NJ). This systematic review was conducted and reported in accordance with the PRISMA guidelines [47].

## Results

### Study characteristics

**Fig. 1:**
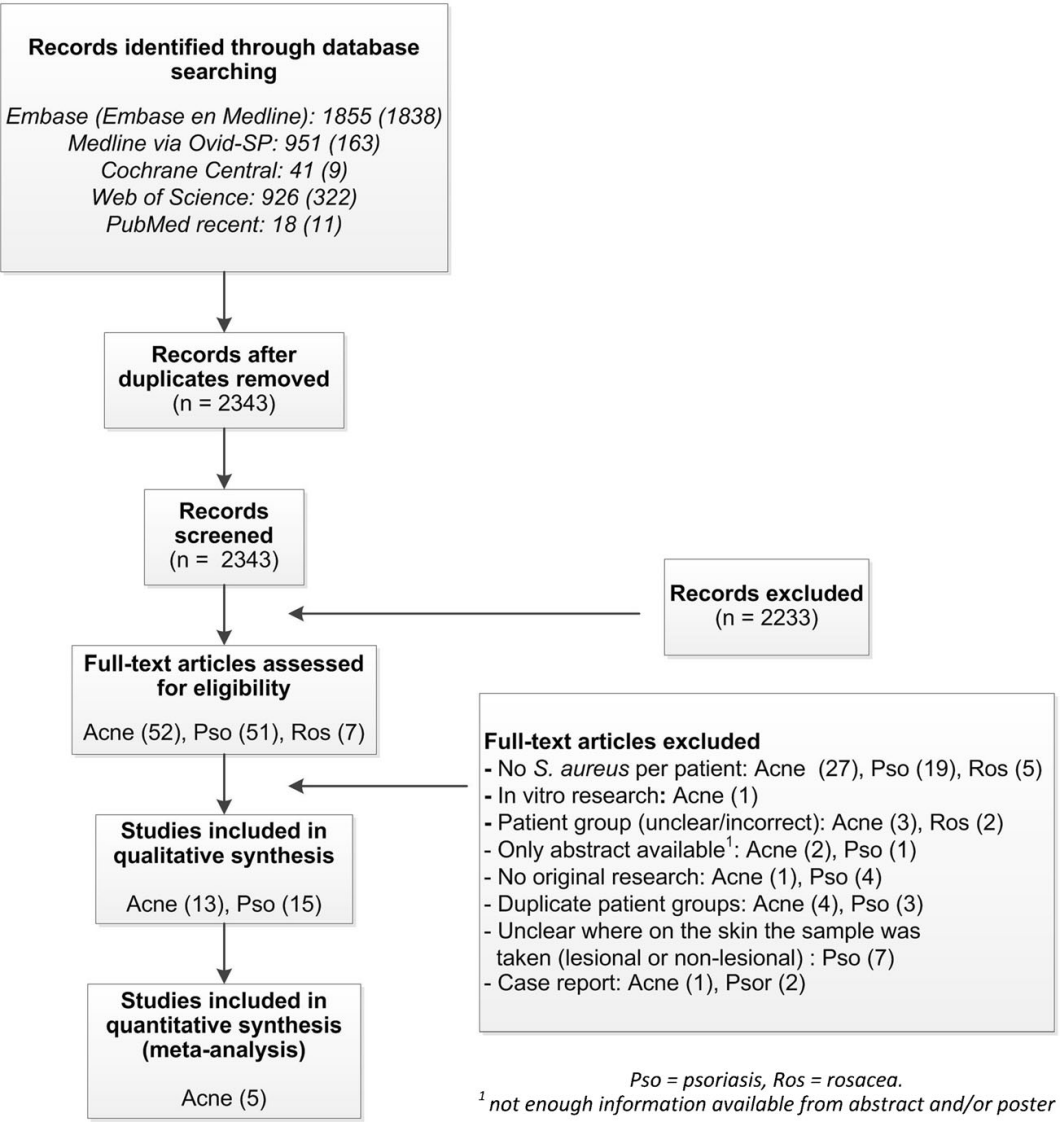
Flow chart of search strategy and study selection

The search yielded a total of 3,791 articles. After deduplication 2,343 articles remained. Based on title and abstract we identified 51 references on psoriasis, 52 on acne and seven on rosacea (Fig. 1). Twenty-eight references, 15 about psoriasis and 13 about acne, met our inclusion criteria after reading the full text. All studies had an observational study design. No study in patients with rosacea met our inclusion criteria. Methods to collect and identify *S. aureus* and study characteristics are described in Table S2a and S2b.

### Quality of the studies

We rated the quality of the included articles with a control group as fair (n = 6) and poor (n = 6). The quality of the uncontrolled studies varied from 0 to 6 points of 7 on the NOS. Table S3a and S2b describe the NOS scores per study. The main reason for downgrading the quality of controlled studies was incomparability of the patient and control groups. Reasons for downgrading the uncontrolled studies were limited description of the methods used for collection of bacteria and identification of *S. aureus*. Low NOS scores are also partly due to inclusion of abstracts, describing limited information on methods. Selection bias might have occurred as the studies included in the review often concern a very specific disease population (treated mostly in tertiary centres). Furthermore, the impact of exposures such as treatment regimen at the moment of collection was poorly reported, which might have resulted in performance bias.

### Colonization with S. aureus of skin and mucosae in psoriasis

Eight of the 15 studies about psoriasis examined *S. aureus* on lesional skin. The proportion of patients with *S. aureus* on lesional skin varied from 0.03 (95 % CI 0.02–0.06) to 0.64 (95 % CI 0.50–0.76) (Table 1). Three of these studies included a control group and one showed a significant increase of colonization of the skin in patients compared to controls (odds ratio [OR] 18.86; 95 % CI 2.20–161.99).

**Table 1:** Event rates and odds ratios of skin colonization with *S. aureus* in psoriatic patients and controls

Author		Patients			Controls		Odds ratio (95 % CI)
		Sample size	Event rate colonization lesional skin (95 % CI)	Event rate colonization non-lesional skin (95 % CI)	Sample size	Event rate colonization skin (95 % CI)	
Atefi et al. 2012 [19]		40	0.08 (0.02–0.21)		40	0.03 (0.00–0.16)	3.16 (0.32–31.78)
Tabatabaei et al. 2011 [48]		50		0.06 (0.02–0.17)	50	0.02 (0.00–0.13)	3.13 (0.31–31.14)
Balci et al. 2009 [13]		50	0.64 (0.50–0.76)	0.14 (0.07–0.27)			
Tomi et al. 2005 [49]		25	0.44 (0.26–0.63)		25	0.04 (0.01–0.24)	18.86 (2.20–161.99)
Ryu et al. 2003 [50]		22	0.45 (0.26–0.65)	0.33 (0.17–0.55)^a^	25	0.32 (0.17–0.52)	Lesional skin 1.74 (0.53–5.70) Non-lesional 1.04 (0.31–3.56)
Brook et al. 1999 [51]		28	0.54 (0.35–0.71)				
Sayama et al. 1998 [52]		100	0.15 (0.09–0.23)	0.06 (0.02–0.13)^b^			
Noah et al. 1990 [53]	Overall ^c^	297	0.03 (0.02–0.06)				
	Axilla	297	0.01 (0.00–0.03)				
	Submammary	297	0.01 (0.00–0.03)				
	Umbilical	297	0.02 (0.01–0.05)				
	Inguinal	297	0.02 (0.01–0.05)				
	Gluteal	297	0.10 (0.07–0.14)				
	Vaginal	297	0.03 (0.01–0.05)				
	Penis-scrotal	297	0.04 (0.02–0.07)				
Weissmann et al. 1980 [54]	Overall ^d^	10		0.55 (0.42–0.67)			
	Arm before treatment	10		0.50 (0.22–0.78)			
	Back before treatment	10		0.60 (0.30–0.84)			
	Arm after treatment	10		0.40 (0.16–0.70)			
	Back after treatment	9		0.33 (0.11–0.66)			
Aly et al. 1976 [55]		40	0.20 (0.10–0.35)	0.13 (0.05–0.27)			

^a^ Sample size is 21

^b^ Sample size is 89

^c^ Average event rate of the different locations on the skin

^d^ Average event rate of multiple locations on the skin before treatment

Non-lesional skin was examined in six studies. The prevalence of patients with colonization varied between 0.06 (95 % CI 0.02–0.13) and 0.55 (95 % CI 0.42–0.67) (Table 1). The two studies that included a control group did not find a statistically significant difference in colonization between psoriatic patients and healthy controls (Tabatabaei 2011; OR 3.13; 95 % CI 0.31–31.14 and Ryu 2003; OR 1.04; 95 % CI 0.31–3.56) [48, 50].

Seven studies evaluated nasal colonization with the proportion of patients with nasal colonization varying from 0.27 (95 % CI 0.13–0.49) to 0.76 (95 % CI 0.62–0.86) (Table 2). Five studies included a control group of which three reported a statistically significant increased nasal colonization rate in patients with psoriasis compared to healthy controls. ORs varied from 1.73 (95 % CI 1.16–2.58) to 14.64 (95 % CI 2.82–75.95) (Table 2).

**Table 2:** Event rates and odds ratios of nasal colonization with *S. aureus* in psoriatic patients and controls

Author	Patients		Controls		Odds ratio (95 % CI)
	Sample size	Event rate of nasal colonization (95 % CI)	Sample size	Event rate of nasal colonization (95 % CI)	
Andersen et al. 2013 [56]	112	0.36 (0.28–0.45)	1985	Not mentioned	1.73 (1.16–2.58)
Balci et al. 2009 [13]	50	0.50 (0.36–0.64)	50	0.34 (0.22–0.48)	1.94 (0.87–4.35)
Tomi et al. 2005 [49]	25	0.56 (0.37–0.74)	25	0.08 (0.02–0.27)	14.64 (2.82–75.95)
Ryu et al. 2003 [50]	22	0.27 (0.13–0.49)	25	0.24 (0.11–0.44)	1.18 (0.32–4.40)
Klein et al. 1997 [57]	33	0.33 (0.19–0.51)			
Singh et al. 1978 [58]	50	0.76 (0.62–0.86)	33	0.6 (0.43–0.75)	2.64 (1.03–6.79)
Aly et al. 1976 [55]	40	0.30 (0.18–0.46)			

A meta-analysis was not performed as the treatment regimen was not adequately described or differed between the studies.

Three studies measured *S. aureus* colonization in the pharynx. The proportion of patients with pharyngeal colonization was 0.04 (95 % CI 0.03–0.07) (Noah 1990), 0.50 (95 % CI 0.30–0.70) (Ajib 2005) and 0.20 (95 % CI 0.13–0.29) (Sayama 1998)) [52, 53, 59]. The presence of *S. aureus* toxins was investigated in seven studies. An overview of the results of these studies can be found in Table S4. Balci et al. concluded that psoriatic patients were colonized with toxigenic strains more frequent than controls (p = 0.006) [13]. Ajib et al. and Tabatabaei et al. also show data that support a role of toxins in lesions but no statistically significant difference between patients and controls was reported [48, 59]. Tomi et al. found a statistically significant relation between a higher Psoriasis Area Severity Index (PASI) score and enterotoxin-positive *S. aureus* versus toxin negative *S. aureus* (p = 0.001) [49].

### Colonization with S. aureus of skin and mucosae in acne

**Fig. 2:**
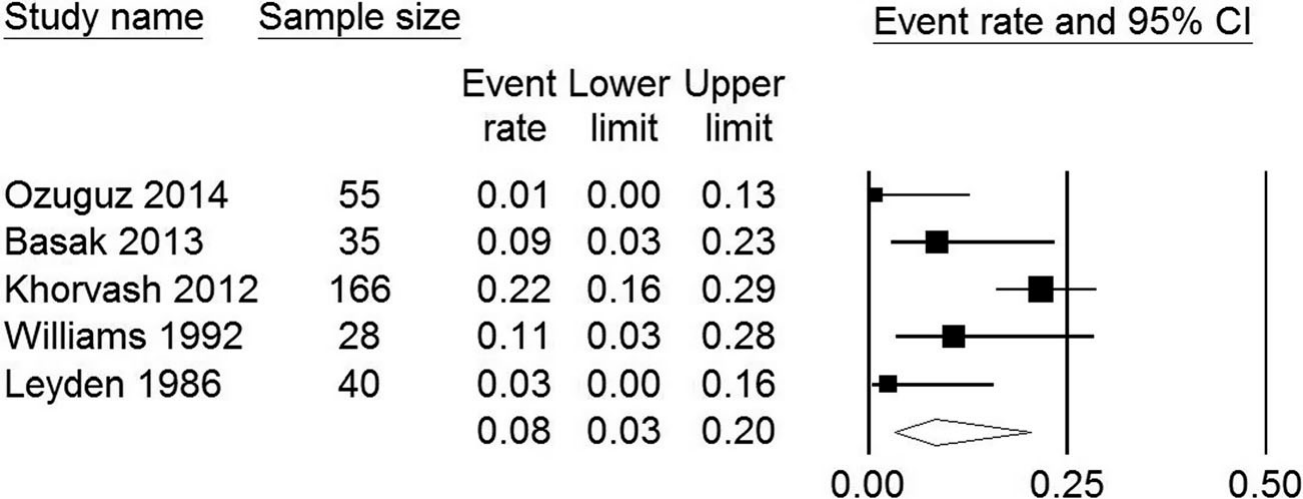
Forest plot of studies reporting proportions of acne patients with nasal *S. aureus* colonization receiving no treatment at the time of sampling. I^2^ = 71

Seven of the 13 articles evaluating acne patients reported on lesional skin colonization with *S. aureus*. The proportion of patients with skin colonization varied between 0.01 (95 % CI 0.00–0.07) and 0.54 (95 % CI 0.40–0.67) (Table 3). Three studies compared skin colonization between patients and controls. Only one found a significant OR of 4.16 (95 % CI 1.74–9.94) (Table 3) [60, 62, 65]. Seven studies reported on the prevalence of nasal *S. aureus* colonization (Table 4). Pooled analysis from the five studies in which patients were not on antimicrobial treatment (324 patients) showed a nasal colonization rate of 0.08 (95 % CI 0.03–0.20) (Fig. 2). With an I^2^ of 71 %, heterogeneity was considered to be moderate. Two studies included a control group but did not demonstrate a statistically significant difference in *S. aureus* nasal colonization rates between patients and healthy controls [66, 69]. Basak et al., Fanelli et al. and Levy et al. found pharyngeal colonization rates of 0.09, 0.33 and 0.26, respectively [67, 70, 71]. No studies reported on the relation between severity of acne and colonization with *S. aureus* or presence of *S. aureus* virulence factors.

**Table 3:** Event rates and odds ratios of skin colonization with S. aureus in acne patients and controls

Author		Patients			Controls		Odds ratio (95 % CI)
		Sample size	Event rate of lesional skin colonization (95 % CI)	Event rate of non-lesional skin colonization (95 % CI)	Sample size	Event rate skin colonization (95 % CI)	
Numata et al. 2013 [60]		100	0.01 (0.00–0.07)^a^		100	0.01 (0.00–0.07)^a^	
Moon et al. 2012 [61]		100	0.06 (0.03–0.13)				
Hassanzadeh et al. 2008 [62]		100	0.41 (0.32–0.51)		100	0.30 (0.22–0.40)^b^	1.62 (0.90–2.91)
Williams et al. 1992^c^ [63]	Before treatment	28	0.02 (0.00–0.22)^a^				
	1 month after start	28	0.14 (0.05–0.32)				
	2 months after start	28	0.32 (0.18–0.51)				
	3 months after start	28	0.21 (0.10–0.40)				
	4 months after start	28	0.25 (0.12–0.44)				
	5 months after start	28	0.02 (0.00–0.22)^a^				
Al Mishari et al. 1987^d^ [35]	Before treatment	20	0.05 (0.01–0.28)	0.05 (0.01–0.28)			
	4 months after start	20	0.02 (0.00–0.29)^a^				
Leyden et al. 1986^e^ [64]	Before treatment	40	0.03 (0.00–0.16)				
	1 month after start	40	0.15 (0.07–0.30)				
	5 months after start	40	0.50 (0.35–0.65)				
Batova et al. 1971 [65]	Swab without buffer	40	0.15 (0.07–0.30)				
	Swab with buffer	50	0.54 (0.40–0.67)		50	0.22 (0.13–0.36)	4.16 (1.74–9.94)

^a^ Event rate = 0.00 (OR not calculated)

^b^ Healthy skin of patients is used as a control

^c^ Patients were treated with isotretinoin for 4 months

^d^ Patients were treated with tetracycline for 4 months

^e^ Patients were treated with isotretinoin for 5 months

**Table 4:** Event rates and odds ratios of nasal colonization with *S. aureus* in patients with acne patients and controls

Author		Patients		Controls		Odds ratio
		Sample size	Event rate of nasal colonization (95 % CI)	Sample size	Event rate of nasal colonization (95 % CI)	
Ozuguz et al. 2014^a^ [66]	Before treatment	55	0.01 (0.00–0.13)^e^	20	0.02 (0.01–0.29)^e^	
	3 months after start	55	0.09 (0.04–0.20)			
Basak et al. 2013^b^ [67]	Before treatment	35	0.09 (0.03–0.23)			
	After treatment	35	0.40 (0.25–0.57)			
Toyne et al. 2012 [68]		116	0.26 (0.19–0.35)			
Khorvash et al. 2012 [69]		166	0.22 (0.16–0.29)	158	0.27 (0.20–0.34)	0.77 (0.46–1.29)
Fanelli et al. 2011 [70]		83	0.19 (0.12–0.29)			
Williams et al. 1992^c^ [63]	Before treatment	28	0.11 (0.03–0.28)			
	1 month after start	28	0.32 (0.18–0.51)			
	2 months after start	28	0.57 (0.39–0.74)			
	3 months after start	28	0.46 (0.29–0.64)			
	4 months after start	28	0.32 (0.18–0.51)			
	5 months after start	28	0.43 (0.26–0.61)			
Leyden et al. 1986^d^ [64]	Before treatment	40	0.03 (0.00–0.16)			
	1 months after start	40	0.30 (0.18–0.46)			
	5 months after start	40	0.70 (0.54–0.82)			

^a^ Twenty patients were treated with oral antibiotics, 20 with isotretinoin, 15 patients received no treatment

^b^ Twenty patients were treated with isotretinoin for an unknown duration

^c^ Patients were treated with isotretinoin for 4 months

^d^ Patients were treated with isotretinoin for 5 months

^e^ Event rate = 0.00 (OR not calculated)

## Discussion

In this systematic review 28 observational studies comprising 1,880 patients were included to evaluate skin and mucosal colonization in patients with psoriasis, acne and rosacea. Patients with psoriasis seem to be colonized with *S. aureus* in the nose more often than healthy controls. One study shows an increased risk of skin colonization in patients compared with controls. However, the sample size of this study was rather small (n = 25) [49]. The literature about virulence factors in psoriatic patients versus controls is ambiguous. Only one study reported about disease severity in relation to *S. aureus* and found a statistically significant relation between a higher PASI score and enterotoxin-positive *S. aureus* versus toxin negative *S. aureus*. In studies on acne, an association between skin colonization and the disease was only found in one of the three articles and therefore less evident [60, 62, 65]. In almost one tenth of the patients with acne *S. aureus* was present in the nose. This is low compared with the nasal colonization rate of healthy people (around 25 %) [72]. Although the patients included in the meta-analysis did not use treatment at the time of sampling, the low colonization rates might be due to a long-term effect of former use of antibiotics [70, 71]. Two studies compared nasal colonization in acne patients with healthy controls and found no statistically significant difference. It has to be noted that in all of these studies there were only few discussions on whether the presence of *S. aureus* represented clear colonization or whether the bacteria were causal agents of pathogenicity. No studies could be included that assessed *S. aureus* colonization in patients with rosacea. Also in current review literature *S. aureus* is not implicated in the pathophysiology of rosacea [9]. Limitations in study design, such as incomparability of patient and control groups, as well as indirectness and imprecision due to low sample sizes, rate the quality of the evidence down and should be taken in consideration when interpreting the results. Essential information about factors that influence microbiota such as treatment regimen, the exact skin site where a sample was taken and duration of the disease which might influence the antimicrobial effect of the host immune response, was often missing. This hampered our ability to draw conclusions about the bacterial ecology of the skin [73–75].

Few studies performed a pre- and post treatment analysis of *S. aureus* abundance. Weissmann et al. described a decrease in the percentage of colonized psoriasis patients from 55 % to 36 % after PUVA (photochemotherapy) [54]. Three studies reported an increase of *S. aureus* colonization of nose, oropharynx and skin after isotretinoin therapy for acne (of which one was statistically significant), whereas Ozuguz et al. found no change [63, 64, 66, 67]. In cross-sectional studies Fanelli et al. and Levy et al. demonstrated that antibiotic use decreased the prevalence of *S. aureus* nasal and oropharyngeal colonization [70, 71]. However, an increase of *S. aureus* colonization was seen after tetracycline therapy in Ozuguz et al [66]. None of the above-mentioned studies reported on the relation between changes in *S. aureus* density and clinical symptoms which could give important information with respect to *S. aureus* causality.

This review has some limitations. Determining the prevalence of *S. aureus* colonization was not the primary objective in a substantial part of the studies. Therefore, indirectness of the evidence with regard to the population might have occurred. Some studies comprised small sample sizes resulting in effect estimates with wide confidence intervals. Controlled studies did not adjust for confounders, such as age, which might influence the given odds ratio. There is a possibility of publication bias as only published studies were considered and as literature focuses on just a few of the many toxins that *S. aureus* produces, this review provides a limited insight on the correlation between toxins of *S. aureus* and psoriasis and acne.

Future research should have a more longitudinal character and focus on bacteria as part of an ecosystem related to severity. This might give more insight in the cause–consequence relation between microorganisms and disease. It is important to consider that the capacity of a microbe to promote disease also depends on other factors such as the host’s genetic predisposition, skin barrier integrity and the coexistence of other microbes. As *S. aureus* is common at all depths of the skin, biopsies might be of additional value next to the techniques that evaluate the superficial skin [76]. Molecular approaches to analyse these samples will give a more complete picture of the microbial diversity facilitating the evaluation of low abundance species and their influence [37, 74].

This systematic review summarises all available data on *S. aureus* colonization and the presence of virulence factors on the skin and mucosa of patients with psoriasis and acne. Patients with psoriasis seem to be colonized with *S. aureus* more often than healthy controls. The overabundant *S. aureus* in the microbiome of psoriasis patients might play a role in perpetuating chronic inflammation. For patients with acne a relation between colonization and the disease was less evident and for rosacea no information about colonization could be obtained from the literature. Determining the presence of individual bacterial species with inflammatory potential, including *S. aureus*, in patients compared with controls is a first step towards elucidating their possible role in skin diseases and might lead to new options for more targeted antimicrobial therapy.

## Electronic supplementary material

Below is the link to the electronic supplementary material.

ESM 1 (DOCX 47.3 kb)
