# Supplementary material for: A systematic review and meta-analysis on Staphylococcus aureus carriage in psoriasis, acne and rosacea
Source: Eur J Clin Microbiol Infect Dis. 2016 May 5;35:1069–77. doi: 10.1007/s10096-016-2647-3 (PMC4902839; doi:10.1007/s10096-016-2647-3)
Supplement: Supplementary file 1 — (DOCX 47.3 kb) [file 10096_2016_2647_MOESM1_ESM.docx]

SUPPLEMENTARY MATERIAL: QUALITY ASSESSMENT SCORE
**Extended Newcastle Ottawa Quality assessment scale**

Maximum score of 8. Uncontrolled studies can reach a maximum score of 7.

Selection

1. Representativeness of the exposed cohort
2. Truly representative of the general acne/psoriasis/rosacea population *
3. Somewhat representative of the general acne/psoriasis/rosacea population *
4. Selected group of patients: hospital based, tertiary centre, inpatients, outpatients
5. No description of the derivation of the cohort
6. Selection of the non-exposed cohort
7. Representative of the average community (healthy control, community control)*
8. Selected group of controls (hospital controls, other dermatological condition)
9. No control group or no description of control group
10. Ascertainment the acne/psoriasis/rosacea diagnosis
11. Diagnosed by dermatologist *
12. Diagnosed by physician other than dermatologist*
13. Diagnosed by clinical assessment*
14. Based on self-report
15. No description of acne case definition
16. Assessment of disease severity
17. Disease severity was assessed with a validated score (doctor assessed)*
18. Disease severity was assessed using another score
19. No disease severity reported

Comparability

1. Comparability of patients and healthy controls on the basis of design or analysis
2. Study controls for confounding using a multivariate model*
3. Atopic dermatitis patients and healthy controls are matched (for age and/or gender)*
4. No controlling for confounding or matching

Outcome

1. Assessment of outcome: colonization or presence of virulence factors (measurement)
2. Method of determination was well described o.a. MSA plates, PCR or ELISA*
3. Not mentioned
4. Assessment of outcome: method of sample taking
   a) Method of sample taking was well described*
   b) Not well described or not mentioned
5. Was there treatment during sampling
6. No treatment *
7. Systemic treatment
8. Topical treatment
9. Not mentioned

**Modified Scoring algorithm controlled studies.**

| **Quality rating** | **Points in Selection Domain** | **Points in Comparability Domain** | **Points in Outcome domain** |
| --- | --- | --- | --- |
| Good | ≥ 3 | ≥ 1 | ≥ 2 |
| Fair | 2 | 0 | ≥ 2 |
| Poor | 0-1 | 0 | 0-1 |

**SUPPLEMENTARY TABLES**
Supplementary Table 1. Digital search strategy (last updated on 16^th^ of September 2014)

| **Database** | **Search string** |
| --- | --- |
| Embase | ('Staphylococcus aureus'/exp OR 'Staphylococcal skin infection'/de *OR* 'Microbiome'/de OR 'Skin flora'/de OR *'Staphylococcus alpha toxin'/de OR 'Staphylococcus toxin'/de OR 'Staphylococcus enterotoxin'/de OR 'Staphylococcus enterotoxin A'/de OR 'Staphylococcus enterotoxin B'/de OR 'Staphylococcus enterotoxin C'/de OR 'Staphylococcus protein A'/de OR 'Panton Valentine leukocidin'/de OR 'Superantigen'/de OR* (((cutan* OR skin* OR derma* *OR nasal OR nose OR nare* OR mucos**) NEAR/3 (flora* OR microflora* OR microbio* OR bacteri* OR staph*)) OR ((staph* OR S OR St) NEAR/3 (aureus* OR pyogenes))):ab,ti OR (*(staph*:ab,ti OR 'Staphylococcus infection'/exp OR 'Staphylococcus'/exp) AND ('Alpha toxin'/de OR 'Bacterial toxin'/de OR 'Exfoliatin'/de OR 'Leukocidin'/de OR 'Leukotoxin'/de OR 'Bacterial antigen'/de OR 'Cytotoxin'/de OR 'Enterotoxin'/de OR 'Hemolysin'/de OR 'Exotoxin'/de OR (superantigen* OR toxin* OR cytotoxin* OR hemoly* OR haemoly* OR enterotoxin* OR exotoxin* OR exfoliatin* OR leucotoxin* OR leukotoxin* OR leukocidin* OR leucocidin* OR epidermoly* OR dermoly**):ab,ti))) AND ('Acne'/exp OR 'Rosacea'/exp OR 'Psoriasis'/exp OR (acne* OR rosacea* OR rhinophyma* OR psoria* OR (Andrew* NEAR/3 disease*) OR ((pustulos* OR bacterid*) NEAR/3 (palm* OR planta* OR sole*))):ab,ti) *NOT ([animals]/lim NOT [humans]/lim)* |
| Medline via OvidSP | (exp "Staphylococcus aureus"/ OR "Staphylococcal skin infections"/ *OR exp* "Microbiota"/ OR (*staphylococcal alpha toxin OR enterotoxin A, staphylococcal OR enterotoxin B, staphylococcal OR enterotoxin C, staphylococcal OR enterotoxin D, staphylococcal OR enterotoxin E, staphylococcal OR enterotoxin G, staphylococcal OR enterotoxin I, staphylococcal OR staphylococcal enterotoxin J OR staphylococcal enterotoxin H OR SEIO enterotoxin, Staphylococcus aureus OR SEIM enterotoxin, Staphylococcus aureus OR Hlb protein, Staphylococcus aureus OR Gamma-hemolysin, Staphylococcus aureus OR Panton-Valentine leukocidin).mp. OR "Staphylococcal protein A"/ OR*  *"Superantigens"/ OR* (((cutan* OR skin* OR derma* *OR nasal OR nose OR nare* OR mucos**) ADJ3 (flora* OR microflora* OR microbio* OR bacteri* OR staph*)) OR ((staph* OR S OR St) ADJ3 (aureus* OR pyogenes))).ab,ti. OR (*(staph*.ab,ti. OR "Staphylococcal infections"/ OR "Staphylococcus"/) AND ("Bacterial toxins"/ OR "Leukocidins"/ OR "Leucocidins"/ OR leukotoxin.mp. OR "Antigens, Bacterial"/ OR exp "Cytotoxins"/ OR "Enterotoxins"/ OR "Hemolysin Proteins"/ OR exp "Exotoxins"/ OR (superantigen* OR toxin* OR cytotoxin* OR hemoly* OR haemoly* OR enterotoxin* OR exotoxin* OR exfoliatin* OR leucotoxin* OR leukotoxin* OR leukocidin* OR leucocidin* OR epidermoly* OR dermoly**).ab,ti.))) AND ("Acne Vulgaris"/ OR exp "Rosacea"/ OR "Psoriasis"/ OR (acne* OR rosacea* OR rhinophyma* OR psoria* OR (Andrew* ADJ3 disease*) OR ((pustulos* OR bacterid*) ADJ3 (palm* OR planta* OR sole*))).ab,ti.) *NOT (animals NOT humans).sh.* |
| Web of Science | TS=((((cutan* OR skin* OR derma* *OR nasal OR nose OR nare* OR mucos**) NEAR/3 (flora* OR microflora* OR microbio* OR bacteri* OR staph*)) OR ((staph* OR S OR St) NEAR/3 (aureus* OR pyogenes)) OR (*staph* AND (superantigen* OR toxin* OR cytotoxin* OR hemoly* OR haemoly* OR enterotoxin* OR exotoxin* OR exfoliatin* OR leucotoxin* OR leukotoxin* OR leukocidin* OR leucocidin* OR epidermoly* OR dermoly**))) AND (acne* OR rosacea* OR rhinophyma* OR psoria* OR (Andrew* NEAR/3 disease*) OR ((pustulos* OR bacterid*) NEAR/3 (palm* OR planta* OR sole*))) NOT ((animal* OR pig* OR sheep* OR horse*) NOT (human* OR patient*))) |
| Cochrane Central | ((((cutan* OR skin* OR derma* *OR nasal OR nose OR nare* OR mucos**) NEAR/3 (flora* OR microflora* OR microbio* OR bacteri* OR staph*)) OR ((staph* OR S OR St) NEAR/3 (aureus* OR pyogenes))):ab,ti OR (*staph*:ab,ti AND ((superantigen* OR toxin* OR cytotoxin* OR hemoly* OR haemoly* OR enterotoxin* OR exotoxin* OR exfoliatin* OR leucotoxin* OR leukotoxin* OR leukocidin* OR leucocidin* OR epidermoly* OR dermoly**):ab,ti))) AND ((acne* OR rosacea* OR rhinophyma* OR psoria* OR (Andrew* NEAR/3 disease*) OR ((pustulos* OR bacterid*) NEAR/3 (palm* OR planta* OR sole*))):ab,ti) |
| Pubmed | (((cutan*[tiab] OR skin*[tiab] OR dermal[tiab] OR dermatol*[tiab] *OR nasal[tiab] OR nose[tiab] OR nare*[tiab] OR mucos*[tiab]*) AND (flora*[tiab] OR microflora*[tiab] OR microbio*[tiab] OR bacterial[tiab] OR bacterio*[tiab] OR staph*[tiab])) OR ((staph*[tiab] OR S[tiab] OR St[tiab]) AND (aureus*[tiab] OR pyogenes[tiab])) OR (*staph*[tiab] AND (superantigen*[tiab] OR toxin*[tiab] OR cytotoxin*[tiab] OR hemoly*[tiab] OR haemoly*[tiab] OR enterotoxin*[tiab] OR exotoxin*[tiab] OR exfoliatin*[tiab] OR leucotoxin*[tiab] OR leukotoxin*[tiab] OR leukocidin*[tiab] OR leucocidin*[tiab] OR epidermoly*[tiab] OR dermoly*[tiab]*))) AND (acne*[tiab] OR rosacea*[tiab] OR rhinophyma*[tiab] OR psoria*[tiab] OR Andrew*[tiab] OR pustulosis palm*[tiab] OR pustular bacterid*[tiab] OR palmoplantar pustulos*[tiab]) AND publisher[sb] |

Supplementary Table 2a. Characteristics of the studies about psoriasis included in the review.

| **Author** | **Country** | **Patients with psoriasis** | | | | | **Healthy controls** | | | **Collection method** | **Detection method** | **NOS** |
| --- | --- | --- | --- | --- | --- | --- | --- | --- | --- | --- | --- | --- |
|  |  | **N** | **% male** | **Range age** | **Treatment at baseline** | **Type** | **N** | **% male** | **Range age** |  |  |  |
| Andersen 2013^4^ [55] | Denmark | 112 | - | - | - | - | 1985 | - | - | swab | colony morphology and catalase test | 4/8 Fair |
| Atefi 2012 [19] | Iran | 40 | 65 | 9-86 | oral or topical antibiotics or PUVA therapy two weeks before study excluded. | plaque | 40 | 65 | 39^1^ | cotton applicator with 0.15 M NaCl | PCR | 4/8 Poor |
| Tabatabaei 2011^3^ [47] | Iran | 50 | - | - | - | - | 50 | - | - | biopsy | PCR (Roche, High Pure DNA ExtractionKit & Bioneer primer) | 2/8 Poor |
| Balci 2009^3^ [13] | Turkey | 50 | - | - | - | chronic plaque | 50 | sex and age matched | | - | - | 3/8  Poor |
| Tomi 2005 [48] | Austria | 25^2^ | 72 | 21-80 | Treatment used: topical corticosteorids, vitamin D analogue calcipotriene, ointments containing salicylic acid and psoralen UVA- therapy or combination. | - | 25 | 48 | 20-58 | swab | coagulase test, Staphylococcal enterotoxin test –reversed passive latex agglutination test | 5/8 Fair |
| Ajib 2005 [58] | Lebanon | 22 | - | - | - | 10 guttate, 9 vulgaris, 3 pustular | 22 | - | - | swab | coagulase test, SET RPLAR test, PCR | 3/8 Poor |
| Ryu 2003 [49] | South Korea | 22 | - | - | - | - | 25 | - | - | skin: tape method nares: swab | coagulase test | 4/8 Fair |
| Brook 1999 [50] | USA | 28 |  |  | Exclusion: systemic or local treatment with antibacterial of antifungal medication | secondarily infected pustular |  |  |  | swab | ‘culture’ | 2/7 |
| Sayama 1998 [51] | Japan | 100 | 56 | 18-84 | - | chronic plaque |  |  |  | swab | coagulase test and Western blot | 2/7 |
| Klein 1997 [56] | USA | 33^2^ | - | - | - | - |  |  |  | swab | - | 1/7 |
| Leung 1991^3,5^ | USA | 11^2^ |  |  |  |  |  |  |  | - | ELISA | 1/7 |
| Noah 1990 [52] | USA | 297 | - | - | Exclusion patients receiving systemic and/or topical antibiotics for a month. Tar treatment stopped 3 days before study. | - |  |  |  | a.o. smears | (culture) | 0/7 |
| Weissmann 1980 [53] | UK | 10 | 60 | 20-75 | No topical steroids, no dithranol | chronic plaque |  |  |  | detergent scrub method | coagulase and DNase test | 2/7 |
| Singh 1978 [57] | India | 50 | - | - | No internal or external treatment 10 days before |  | 33 | - | - | Detergent scrub method | coagulase test | 2/8 Poor |
| Aly 1976 [54] | USA | 40 | - | - | - | - |  |  |  | detergent scrub method | colony morphology on selective media | 2/7 |
| - = not mentioned ; 1= only mean age reported; 2= subgroup; 3= only abstract/poster available; 4= diagnosis psoriasis was patient reported; 5= Leung DYM, Reiser RF, Harbeck R, Middleton M, Walsh P, Hanifin JM, Norris DA (1991) Staphylococcal Aureus Grown from the Skin of Atopic-Dermatitis (Ad) and Psoriasis Patients (Pts) Secrete Toxins with Superantigenic Properties. Clin Res 39(2):A422–A422twb .27wtlsb -0.18pt | | | | | | | | | | | | |

Supplementary Table 2b. Characteristics of the studies about acne included in the review

| **Author** | **Country** | **Patients with acne** | | | | **Healthy controls** | | | **Collection method** | **Detection method** | **NOS** |
| --- | --- | --- | --- | --- | --- | --- | --- | --- | --- | --- | --- |
|  |  | **N** | **% male** | **Range age** | **Treatment at baseline** | **N** | **% male** | **Range age** |  |  |  |
| Ozuguz 2014 [65] | Turkey | 55 | 33 | 22 ± 4.2^1^ | No | 20 | - | 22 ± 7.6 | swab | coagulase test | 6/8 Fair |
| Numata 2013 [59] | Japan | 100 | 33 | 14-34 | No topical or systemic corticost. Subgroup antibiotics | 28 | 42 | 15-39 | swab | MSA medium, coagulase test | 5/8  Fair |
| Basak 2013 [66] | Turkey | 35 | 31 | 15-24 | No |  |  |  | swab | MSA medium, coagulase test | 5/7 |
| Toyne 2012 [67] | Australia | 116 | 47 | 14-40 | Amongst others antibiotics |  |  |  | swab | (culture) | 4/7 |
| Moon 2012 [60] | Korea | 100 | 46 | 10-40 | 73 topical or syst AB  27 no treatment |  |  |  | via comedone extractor | Vitek-2 system | 4/7 |
| Khorvash 2012 [68] | Iran | 116 | - | 18-30 | No | 158 | - | 24 ± 3.2^1^ | swab | MSA medium, coagulase test, DNase test | 6/8 Fair |
| Fanelli 2011 [69] | USA | 83 | 38 | 26 ± 9,5^1^ | 34% topical AB, 28% oral |  |  |  | swab | differential *S. aureus* plate (CHROMagar ^TM^) | 3/7 |
| Hassanzadeh 2008 [61] | Iran | 100 | 36 | 18-24 | - | Healthy skin of patients used as control |  |  | - | MSA medium and coagulase tests | 2/7 |
| Levy 2003 [70] | USA | 105 | 30 | 24.5 ± 7.5^1^ | 40% topical and/or oral AB |  |  |  | swab | colony morphology and coagulase test | 4/7 |
| Williams 1992 [62] | UK | 30 | 60 | 15-34 | - |  |  |  | swab | (culture) | 1/7 |
| Al Mishari 1987 [35] | Kuwait | 20 | 60 | 15-28 | - |  |  |  | swab | colony morphology | 2/7 |
| Leyden 1986 [63] | USA | 40 | - | - | No therapy in one month before start study |  |  |  | skin: scrub nose: swab | MSA medium,  Api staph identification system | 3/7 |
| Batova 1971 [64] | Poland | 90 | - | - | - | 50 | - | 18-25 | 2 types of wet swabs | (culture) | 2/7 |
| 1= only mean age reported; - = not mentioned | | | | | | | | | | | |

Supplementary Table 3a. Risk of bias in studies about psoriasis

| **Risk of bias** | **Selection** | | | | **Comparability** | **Outcome** | | |
| --- | --- | --- | --- | --- | --- | --- | --- | --- |
| **Study** | **Representativeness of the exposed cohort for the population** | **Representativeness of the non-exposed cohort for the population** | **Ascertainment of the diagnosis** | **Assessment of disease severity through validated score?** | **Comparability exposed and non-exposed cohort** | **Detection method adequately defined** | **Collection method adequately defined** | **Treatment during measurement of outcome?** |
| Andersen 2013 [55] | x | x | 0 | 0 | 0 | x | x | 0 |
| Atefi 2012 [19] | 0 | 0 | X | 0 | x | x | x | 0 |
| Tabatabaei 2011 [47] | 0 | 0 | 0 | 0 | 0 | x | x | 0 |
| Balci 2009 [13] | 0 | x | 0 | x | x | 0 | 0 | 0 |
| Tomi 2005 [48] | 0 | x | X | x | 0 | x | x | 0 |
| Ajib 2005 [58] | 0 | 0 | 0 | x | 0 | x | x | 0 |
| Ryu 2003 [49] | 0 | x | 0 | x | 0 | x | x | 0 |
| Brook 1999 [50] | 0 | 0 | 0 | 0 | NA | x | x | 0 |
| Sayama 1998 [51] | 0 | 0 | 0 | 0 | NA | x | x | 0 |
| Klein 1997 [56] | 0 | 0 | 0 | 0 | NA | 0 | x | 0 |
| Leung 1991^1^ | 0 | 0 | 0 | 0 | NA | 0 | x | 0 |
| Noah 1990 [52] | 0 | 0 | 0 | 0 | NA | 0 | 0 | 0 |
| Weissmann 1980 [53] | 0 | 0 | 0 | 0 | NA | x | x | 0 |
| Singh 1978 [57] | 0 | 0 | 0 | 0 | 0 | x | x | 0 |
| Aly 1976 [54] | 0 | 0 | 0 | 0 | NA | x | x | 0 |
| ^1^ see supplementary table 2a for reference | | | | | | | | |

Supplementary Table 3b. Risk of bias in studies about acne

| **Risk of bias** | **Selection** | | | | **Comparability** | **Outcome** | | |
| --- | --- | --- | --- | --- | --- | --- | --- | --- |
| **Study** | **Representativeness of the exposed cohort for the population** | **Representativeness of the non-exposed cohort for the population** | **Ascertainment of the diagnosis** | **Assessment of disease severity through validated score?** | **Comparability exposed and non-exposed cohort** | **Detection method adequately defined** | **Collection method adequately defined** | **Treatment during measurement of outcome?** |
| Ozuguz 2014 [65] | 0 | x | 0 | x | x | x | x | x |
| Numata 2013 [59] | 0 | x | X | x | 0 | x | x | 0 |
| Basak 2013 [66] | 0 | 0 | X | x | NA | x | x | x |
| Toyne 2012 [67] | X | 0 | X | x | NA | 0 | x | 0 |
| Moon 2012 [60] | 0 | 0 | X | x | NA | x | x | 0 |
| Khorvash 2012 [68] | 0 | 0 | X | x | x | x | x | x |
| Fanelli 2011 [69] | 0 | 0 | X | 0 | NA | x | x | 0 |
| Hassanzadeh 2008 [61] | x | 0 | 0 | 0 | x | x | 0 | 0 |
| Levy 2003 [70] | 0 | 0 | X | 0 | NA | x | x | x |
| Williams 1992 [62] | 0 | 0 | 0 | 0 | NA | 0 | x | 0 |
| Al Mishari 1987 [35] | 0 | 0 | 0 | 0 | NA | x | x | 0 |
| Leyden 1986 [63] | 0 | 0 | 0 | 0 | NA | x | x | x |
| Batova 1971 [64] | 0 | x | 0 | 0 | 0 | 0 | x | 0 |

Supplementary Table 4: Presence of toxins on skin or mucosa in patients with psoriasis (n/total patients)

| **Author** | **Location and person** | **Staphylococcal enterotoxin (SE) A** | **SEB** | **SEC** | **SED** | **SEE** | **Toxic shock syndrome toxin (TSST) 1** | **Exfoliative toxin** | **Total** |
| --- | --- | --- | --- | --- | --- | --- | --- | --- | --- |
| Atefi 2012 [19] | lesions of patient | 2/3 |  | 3/3 |  |  | 1/3 |  | 3/3 |
|  | nevi of control | 0/1 |  | 1/1 |  |  | 1/1 |  | 1/1 |
| Tabatabaei 2011 [47] | skin patients and nevi control | 2/4 |  | 4/4 | 0/4 |  | 2/4 |  | 4/4 |
| Balci 2009^1^ [13] | lesional skin patient |  |  |  |  |  |  |  | 31/32 |
|  | non-lesional skin patient |  |  |  |  |  |  |  | 3/7 |
|  | nares patient |  |  |  |  |  |  |  | 24/25 |
|  | nares control |  |  |  |  |  |  |  | 7/17 |
| Tomi 2005 [48] | lesional skin and nares patient | 1/15 | 5/15 | 3/15 | 2/15 |  |  |  | 9/15 |
|  | volar site of elbow and nares control | 0/3 | 0/3 | 0/3 | 0/3 |  |  |  | 0/3 |
| Ajib 2005 [58] | pharynx patient | 6/11 | 0/11 | 5/11 | 0/11 |  |  |  | 9/11 |
|  | pharynx control | 0/11 | 0/11 | 0/11 | 0/11 |  |  |  | 0/11 |
| Sayama 1998^2^ [51] | lesional patient |  | 4/15 |  |  |  | 2/15 | 2/15 | 5/15 |
|  | non-lesional patient |  | 3/5 |  |  |  | 2/5 | 0/5 | 4/5 |
|  | pharynx patient |  | 6/19 |  |  |  | 4/19 | 2/19 | 9/19 |
| Leung 1991^3^ | Skin and nose patient | 4/11 | 1/11 | 0/11 |  |  | 1/11 | 0/11 | 6/11 |
| ^1^ Total is based on SEA, SEB, SEC, SED, SEE ^2^ Results of the Western Blot are reported here  ^3^ See supplementary table 2a for reference  SE = Staphylococcal enterotoxin | | | | | | | | | |
